# *phylopath*: Easy phylogenetic path analysis in R

**DOI:** 10.7717/peerj.4718

**Published:** 2018-04-25

**Authors:** Wouter van der Bijl

**Affiliations:** Department of Zoology, Stockholm University, Stockholm, Sweden

**Keywords:** Phylogenetic path analysis, Evolution, Path analysis, Comparative methods, R package

## Abstract

Confirmatory path analysis allows researchers to evaluate and compare causal models using observational data. This tool has great value for comparative biologists since they are often unable to gather experimental data on macro-evolutionary hypotheses, but is cumbersome and error-prone to perform. I introduce *phylopath*, an R package that implements phylogenetic path analysis (PPA) as described by von Hardenberg & Gonzalez-Voyer (2013). In addition to the published method, I provide support for the inclusion of binary variables. I illustrate PPA and *phylopath* by recreating part of a study on the relationship between brain size and vulnerability to extinction. The package aims to make the analysis straight-forward, providing convenience functions, and several plotting methods, which I hope will encourage the spread of the method.

## Introduction

The comparative method is a critical tool to answer macro-evolutionary questions and has been since the start of evolutionary biology itself (Darwin, 1839). It is often the only way to assess the generality of evolutionary patterns. A drawback of the method is that it is observational, not experimental, and is therefore often said to be unable to evaluate causal mechanisms (Martins, 2000). However, causal models *do* predict correlations between certain variables to exist and other correlations to be absent. It is these predictions that are leveraged in path analysis (Shipley, 2000a), a specific form of structural equation modeling, that uses regression to test these predictions. Specifically, as it is used here, statements can be defined about which variables a causal model predicts to be independent, given certain co-variates, and those independencies can be tested. If they are not independent, i.e., a regression coefficient is significantly different from zero, this can be interpreted as evidence against the causal model.

Consider a minimal example, where A causes B and B causes C, i.e., A → B → C. Since there is no direct causal link between A and C, only through B, this causal model predicts that A and C are independent, given B. This prediction can be tested with the regression model C ∼ B + A, where the coefficient of A is predicted to be close to zero. In other words, no effect of A is expected that is additional to the effect of B, since all causal effects of A on C should be mediated by B. This rationale can be expanded to more complicated scenarios and allow critical assessment of whether data supports a causal model. Similarly, several competing causal models can be compared, and it can be assessed which one is best supported by the data (Shipley, 2000b, 2013; von Hardenberg & Gonzalez-Voyer, 2013). Path analysis is of great potential value to comparative biologists since it allows for better use of observational data and emphasizes a quantitative comparison of competing causal evolutionary hypotheses.

In comparative biology normal regression models cannot be used for path analysis since the assumption of independence of observations is violated, as closely related species are expected to be more similar (Felsenstein, 1985; Pagel & Harvey, 1991). This similarity by descent can be corrected for with phylogenetic comparative methods and regression analysis can be performed using phylogenetic generalized least-squares (PGLS) models. von Hardenberg & Gonzalez-Voyer (2013) showed that PGLS can be successfully employed to perform confirmatory path analysis, based on the d-separation method by Shipley (2000b), and termed it phylogenetic path analysis (PPA).

By its nature, PPA is complicated, time consuming and error prone. For the worked exercise in the book chapter outlining the method (Gonzalez-Voyer & von Hardenberg, 2014), the reader needs to define a list of 46 total d-separation statements and fit 21 PGLS models, and then compile the results afterwards. This takes a lot of time, requires a lot of code and the number of steps required increases the chance for errors. Moreover, manual procedures such as intermediary rounding of results can in some cases significantly alter the final results. Therefore, I hope that a specialized software implementation will greatly increase the reproducibility of the method, decrease research effort to perform the analysis and encourage the spread of the method by decreasing entry barriers.

## A Worked Example

### Dataset

I will illustrate the use of the package by recreating a small part of the analysis by Gonzalez-Voyer et al. (2016). This study focused on the possible influence of brain size on the vulnerability to extinction in 474 mammalian species. Note that the goal of the analysis presented here is merely instructional; the original paper present a much more thorough analysis and should be used for biological inference.

The data used in the study is included in the package as red_list and red_list_tree. The data includes seven variables, listed in Table 1. Note that the species names are set as rownames and that these names match the tip labels of red_list_tree. This is how the package matches the observations to the phylogeny.

**Table 1:** The seven variables used in the analysis.

Variable	Description
Br	Brain size
B	Body size
P	Population density
L	Litter size
G	Gestation period
W	Weening age
Status	Vulnerability to extinction, as Red list status

### Defining the causal model set

I start out by defining various relationships common to all causal models. I assume that brain size is caused by body size (a result of allometry), gestation length is a causal parent of both litter size and weening age and that body size is a causal parent of population density, since these are all well-established relationships in the literature. I want to control for allometric effects of body size, and therefore include a direct effect of body size on status and an indirect effect through litter size. Additionally I also assume that the population density and life history variables all affect the vulnerability to extinction (which I will refer to as *status*), to limit the number of models that needs testing.

Since I am interested in testing for direct and indirect effects of brain size, I will vary those effects. Following the original authors, when considering indirect effects, brain size is a causal parent of litter size, gestation period and weaning age. When looking at direct effects, brain size is directly causally linked to status. This then leaves me with four causal hypotheses: a null model where brain size is irrelevant, a model with a direct effect, a model with indirect effects and a model with both.

I define these models using the define_model_set() function. I supply a list of formulas for each model, using c(). Formulas should be of the form child ∼ parent, or you can read the ∼ as “caused by,” and describe each path in your model. Multiple children of a single parent can be combined into a single formula: child ∼ parent1 + parent2. The paths that are shared between all models, can be included using the .common parameter. So I define our four models as follows:
**library**(phylopath)
m <- **define_model_set**(
 null = **c**(),
 direct = **c**(Status**∼**Br),
 indirect = **c**(L**∼**Br, G**∼**Br, W**∼**Br),
 both = **c**(Status**∼**Br, L**∼**Br, G**∼**Br, W**∼**Br),
 .common = **c**(Br**∼**B, P**∼**B, L**∼**B**+**G, W**∼**G, Status**∼**P**+**L**+**G**+**W**+**B)
)

**Figure 1:**
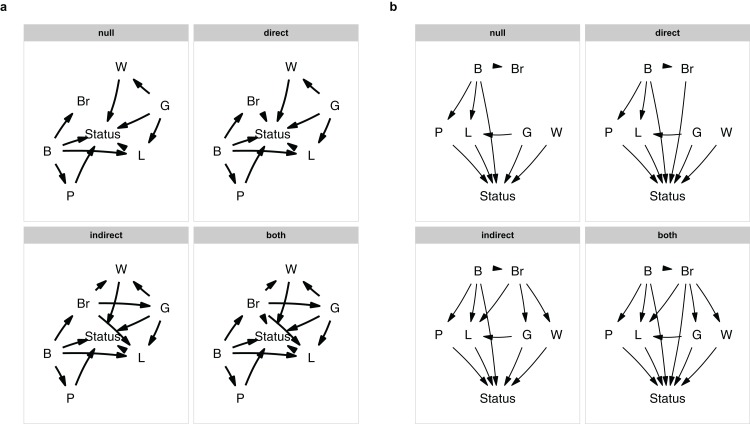
The model set. The model set is laid out algorithmically (A) and manually (B).

It is easy to forget a path, or to make a typo. It is therefore good to make a quick plot to check. You can either plot a single model with, e.g., plot(m$direct), or plot all of them at once (Fig. 1A):
**plot_model_set**(m)

The nodes are laid out algorithmically. I mimic the lay-out used in the paper by manually defining the coordinates in a data.frame (Fig. 1B), which in this case looks much better:
positions <- **data.frame**(
 name = **c**('B', 'Br', 'P', 'L', 'G', 'W', 'Status'),
 x = **c**(2**:**3, **c**(1, 1.75, 3.25, 4), 2.5),
 y = **c**(3, 3, 2, 2, 2, 2, 1)
)
**plot_model_set**(m, manual_layout = positions, edge_width = 0.5)

Defining your model set is perhaps the most crucial part of PPA. Since the method is confirmative and not explorative, you want to strike a good balance between complexity and interpretability.

### Evaluation of the hypotheses

p <- **phylo_path**(m, red_list, red_list_tree)

Printing the result gives us some basic information:

p
## A phylogenetic path analysis, on the variables:
## Continuous: G W B L P Status Br
## Binary:
##
## Evaluated for these models: null direct indirect both
##
## Containing 36 phylogenetic regressions, of which 18 unique

**Figure 2:**
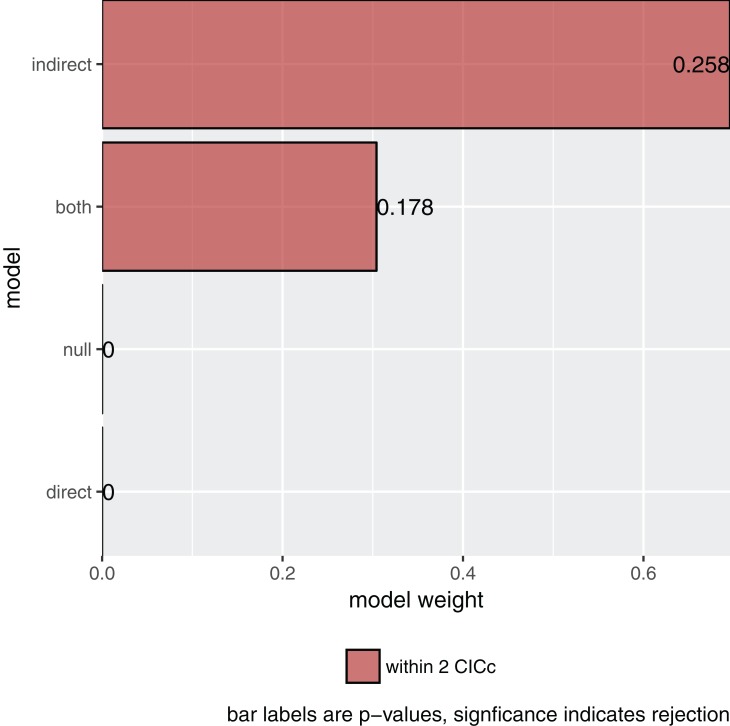
The relative importance of the four causal models.

More importantly, asking for its summary and plotting it (Fig. 2) gives me the actual result of our comparison:
s <- **summary**(p)
s
##   model k q    C   p  CICc delta_CICc   l   w
## 1 indirect 8 20 19.205 0.258 61.059   0.000 1.000 0.696
## 2   both 7 21 18.671 0.178 62.715   1.656 0.437 0.304
## 3   null 11 17 247.625 0.000 282.967  221.908 0.000 0.000
## 4  direct 10 18 247.090 0.000 284.594  223.535 0.000 0.000
**plot**(s)

The summary reports the results table as used by Gonzalez-Voyer & von Hardenberg (2014). Specifically, it reports the model name, the number of independence claims made by the model (*k*), the number of parameters (*q*), the C statistic and the accompanying *p*-value. A significant *p*-value would indicate that the available evidence rejects the model. It also reports model selection information: the C-statistic information criterion corrected for small sample sizes (CICc), the difference in CICc with the top model (delta_CICc) and finally the associated relative likelihoods (*l*) and CICc weights (*w*).

In this example, there is strong support for the indirect pathway. The addition of the direct path in the both model did lead to a small improvement (the C-statistic is lower) but not enough to put it ahead of the indirect model.

### Choosing a final model

So what is the best causal model? Firstly, the null and direct models are not supported since they have significant *p*-values and should therefore be discarded. The indirect pathway is certainly important, but what about the direct pathway? There are several philosophies of dealing with this issue. In this particular case the two top-ranked causal models are directly nested, they share all the same paths except for one. One can think of this like nested regression models. Typically, the extra path should lower the CICc by at least some margin, often two. In this case it does not and I elect to choose the top ranked model (see Arnold, 2010 for a discussion on AIC and uninformative parameters).

After I have found my final model, I can estimate the relative importance of each of the paths. To estimate the paths in the highest ranked model, use the best function:
b <- **best**(p)
**plot**(b, manual_layout = positions)

**Figure 3:**
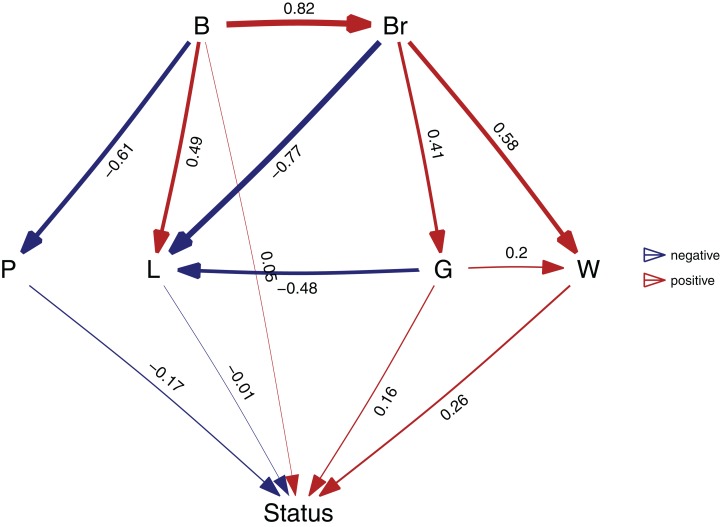
The best supported causal model. A visualization of the best supported causal model, and the standardized path coefficients.

This will return both the standardized regression coefficients, as well as their standard errors. The resulting plot is shown in Fig. 3. In order to get confidence intervals as well, you need to take bootstrap replicates using the boot argument: e.g. b_ci <- best(p, boot = 500), which uses the bootstrap methods of the phylolm package (see “Implementation Notes”). This is disabled by default because it is slow. Using plot will give a visualization of the causal model. You can fit any arbitrary causal model that you evaluated with choice, so in this case choice(p, "both") would give the second ranked model.

**Figure 4:**
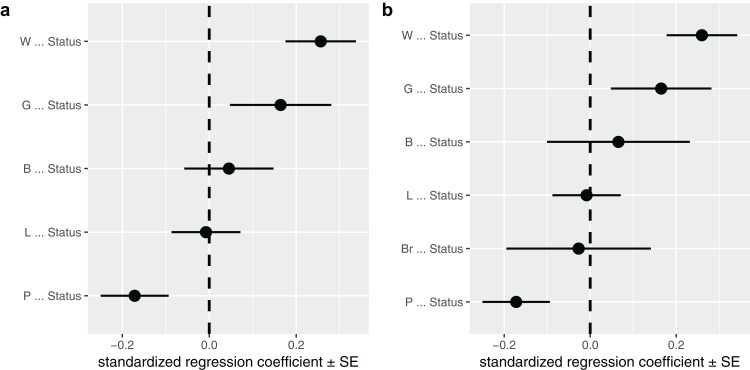
The path coefficients. Standardized path coefficients and their standard errors, for the best supported model (A) and the average of the top two models (B).

A second way to look at a fitted model is to more directly look at the standardized coefficients and errors of the paths using coef_plot. This can be used it to quickly compare the importance of the different variables that affect Status. Although I have modeled five effects on status, they are not necessarily all important and certainly litter size and body size have small effects (Fig. 4A).

**coef_plot**(b, error_bar = "se", order_by = "strength", to = "Status") **+** ggplot2**::coord_flip**()

### Model averaging

In many cases it may not be obvious or correct to choose one model. While in this case the two top competing models were nested, they do not have to be. In cases like these, it may be useful to perform model averaging instead, as discussed and used in the original paper (von Hardenberg & Gonzalez-Voyer, 2013). phylopath makes model averaging easy, and you can quickly average over a selection of the top models, or all models considered. One should take care to not include models with significant C-statistics in the averaging, as these models are not supported. Models are weighted by their likelihood, and these weights can be found in the original summary table in the w column. One needs to choose how to deal with paths that do not occur in all models. One can average path coefficients only between those models that include that path. This is often called conditional averaging and was used by von Hardenberg & Gonzalez-Voyer (2013) and is the default behavior in phylopath. Alternatively, one can consider missing paths to have a coefficient of zero and average over all models, which is often called full averaging. The latter results in *shrinkage*, where the path coefficients that do not occur in all models will shrink toward zero.

In this case, I could choose to average the two competing models. I use full averaging, as I would like uncertain paths to experience shrinkage, and re-evaluate the strength of the coefficients toward Status (Fig. 4B):
avg <- **average**(p, avg_method = "full")
**coef_plot**(avg, error_bar = "se", order_by = "strength", to = "Status") **+** ggplot2**::coord_flip**()

The average function selects the competing models, estimates the standardized path coefficients and then averages them. Note that only the two top models have been averaged, since by default the cut_off is set to two CICc. You can average over all models in the set by using cut_off = Inf (but should only do so when all C-statistics are non-significant, see above).

### Analysis conclusion

A clear rejection of the null model indicates that brain size is related to the vulnerability to extinction of mammals, where large-brained animals high a higher vulnerability. This effect is mediated through life history, where the weaning and gestation periods are more important than litter size. There is no strong evidence in support of a direct effect of brain size on vulnerability to extinction that is independent of life history. The original analysis came to the same conclusion.

## Binary traits

Both continuous and binary data can be included in path analyses performed with phylopath. Practically, this means that some independence statements are tested using linear models (phylolm::phylolm), while others using logistic regression (phylolm::phyloglm), depending on which variable is the dependent variable for that statement (Shipley, 2009). From a user perspective, all one needs do is to make sure your binary variables are of the character of factor class and they will be recognized as binary data. If you have coded your binary variables as numeric zeros and ones, make sure that you convert them first, e.g., using as.factor.

For example, perhaps instead of having actual body sizes, perhaps I only knew whether the animals are small or large. Below I make this new variable, and again run the same phylo_path call as above:
red_list2 <- red_list
red_list2$B <- **ifelse**(red_list$B < 7, "small", "large")
**phylo_path**(m, red_list2, red_list_tree)

Printing now shows:
## A phylogenetic path analysis, on the variables:
## Continuous: G W L P Status Br 
## Binary:   B 
## 
## Evaluated for these models: null direct indirect both 
## 
## Containing 36 phylogenetic regressions, of which 18 unique

This confirms that body size is now modeled as a binary variable. All following analyses will take this into account automatically. Note that path estimates toward binary variables are on a logit scale.

Using a variable with more than two levels is not supported and will result in an error.

## Models of Evolution

phylopath uses the phylolm package in the background (see below) and the models of evolution that are available there are therefore supported. You can simply pass the name of the model of evolution through the model parameter, just like using phylolm directly. It should be noted though, that phylopath by default uses Pagel’s lambda model and not Brownian motion, which is the default for phylolm. I strongly recommend all users to evaluate critically what model of evolution they choose to use. Also, the model of evolution is only applied to continuous variables, i.e., using phylolm::phylolm, and not to binary variables which use phylolm::phyloglm. For the latter, one can choose between the two computational implementations, using the method parameter. When you supply the model or method parameter (or any other modelling parameters through the ellipses: …) to phylo_path, these settings are automatically passed down to other functions, so best, choice, and average all use the same settings to guarantee consistency.

The estimated phylogenetic parameter can be found in the d_sep tables returned by phylo_path in the phylo_par column (you can also see which independence statements are rejected by looking at the *p*-values). For example, one can see the estimates of *lambda* for the null model above:
p**$**d_sep**$**null
## # A tibble: 11 x 4
##               d_sep     p phylo_par    model
##               <chr>    <dbl>  <dbl>    <list>
## 1              G ∼ B 4.634545e-23 0.9828841 <S3: phylolm>
## 2            P ∼ B + G 7.956106e-01 0.7857394 <S3: phylolm>
## 3           G ∼ B + Br 1.931013e-05 0.9782140 <S3: phylolm>
## 4           W ∼ G + B 3.919012e-15 0.9137296 <S3: phylolm>
## 5         W ∼ G + B + L 8.258827e-03 0.9159247 <S3: phylolm>
## 6         P ∼ G + B + W 2.765852e-01 0.7897171 <S3: phylolm>
## 7        W ∼ G + B + Br 4.894272e-05 0.9004170 <S3: phylolm>
## 8         P ∼ G + B + L 9.787521e-01 0.7856850 <S3: phylolm>
## 9         L ∼ G + B + Br 4.993803e-05 0.8878005 <S3: phylolm>
## 10          P ∼ B + Br 1.451390e-01 0.7798783 <S3: phylolm>
## 11 Status ∼ G + W + B + L + P + Br 7.655460e-01 0.2332001 <S3: phylolm>

## Implementation Notes

In addition to the functions outlined above, several lower level functions are also available to the user, specifically est_DAG to estimate the path coefficients of an arbitrary model and average_DAGs to average several fitted models.

phylopath builds on several important packages, a few of which I highlight here. Firstly, it implements PGLS and phylogenetic GLM using phylolm (Ho & Ané, 2014). This implementation was chosen for several reasons, including that the package is fast on large trees, its support for both Gaussian and logistic models and the robust estimation of confidence intervals using bootstrapping.

Furthermore, the ggm (Marchetti, Drton & Sadeghi, 2015) package is used for the ordering of the causal graphs and the finding of the d-separation statements. Model averaging is implemented using the MuMIn (Barton, 2016) package. ape (Paradis, Claude & Strimmer, 2004) is used for checking and pruning phylogenies. ggplot2 (Wickham, 2016) and its ggraph (Pedersen, 2017) extension are used for all plotting methods.

## Conclusion

I have presented phylopath, a package that aims to make PPA more reproducible and less error-prone, and much faster and easier for the analyst. I hope that the package will stimulate the use of PPA amongst evolutionary biologists, as I believe that it is a powerful tool for a field in which experimental data is often impossible to obtain. I welcome bug reports, feedback, and suggestions for the development of phylopath.

## Additional Information and Declarations

### Data Availability

The following information was supplied regarding data availability: All code and data is available in the R package: https://cran.r-project.org/web/packages/phylopath/index.html.
